# Factors Shaping Phenotypic Variation in *Thymus saturejoides*

**DOI:** 10.3390/plants14121772

**Published:** 2025-06-10

**Authors:** Abderrahim Ouarghidi, Imane Abbad, Tiza Mfuni

**Affiliations:** 1 African Studies and Anthropology, Penn State University, State College, PA 16802, USA; 2 Laboratory of Water Sciences, Microbial Biotechnologies, and Natural Resources Sustainability (AQUABIOTECH), Unit of Microbial Biotechnologies, Agrosciences, and Environment (BIOMAGE)-CNRST Labeled Research Unit N°4, Faculty of Sciences-Semlalia, University Cadi Ayyad, P.O. Box 2390, Marrakech 40000, Morocco; 3 Department of Geography, Penn State University, State College, PA 16802, USA

**Keywords:** phenotypic variation, environmental change, *Thymus saturejoides*

## Abstract

Patterns of plant phytochemical composition vary between populations of any plant species and impact the cultural and economic value of important plant species. Phenotypic outcomes are a combination of genetic, environmental, and human influence. *Thymus saturejoides* is endemic to Morocco and Algeria and part of a suite of economically important wild plants used to produce essential oils for the global market in the region. Currently, little is known about the human and ecological factors that shape *T. saturejoides* phenotypic traits. In this paper, we examine the factors that drive phenotypic variation in the species *T. saturejoides* through the chemical composition of essential oil. We used a systematic review protocol to identify 15 published sources, from which we obtained data on chemical composition (secondary metabolites and/or chemotype) for 51 samples, as well as information on the geographic location of harvest listed in the paper. We used the geographic location information to determine elevation, temperature, precipitation, soil type, and soil carbon. We ran linear regression models to determine if any of these environmental variables were associated with the content of key chemicals known to mark quality and value in *T. saturejoides*. Elevation was statistically significant in the models for thymol, linalool, p-cymene, carvacrol (*p* = 0.072), and borneol (*p* = 0.056). Other environmental variables were not statistically significantly related to the content of any of the chemicals. Although we did not find an association between chemical composition and temperature or precipitation, this does not exclude the possibility that a relationship exists at a finer spatial or temporal scale, such as days, weeks, or months. Our findings could also suggest that genetic and human-related factors, such as time of harvest, are more important than environmental factors.

## 1. Introduction

Environmental change is impacting plant diversity and ecosystems worldwide. Not only is climate change causing shifts in species’ distribution range [1], but it is also changing weather patterns in ways that disrupt ecosystems and increase stressors that affect plants’ growth and alter processes associated with chemical composition [2,3,4]. These alterations in the chemical composition of plants will have a significant impact on the economic value of certain plant species. Understanding patterns of plant phytochemical composition in the face of environmental change is, thus, of significant importance to countries and communities whose livelihoods depend on wild plants [5,6]. In Morocco, the harvest of medicinal and aromatic plants (MAPs) is an important source of income for rural communities [7,8], and the production of essential oils for the global market is an important source of revenue for the country [9].

Morocco and the Mediterranean region are already experiencing the impacts of climate change [10,11]. Despite the large body of literature that looks at the impacts of climate change on crop and plant growth and development, very few studies have looked at the impact of climate change on the phytochemical composition of MAP in the Mediterranean area and, specifically, in Morocco. Understanding the drivers of MAP phytochemical composition is an important step toward understanding how climate change might shift the economically important properties of MAP. In this paper, we seek to understand the ways environmental factors shape the phytochemical composition of one of Morocco’s most studied and economically important MAP species: *Thymus saturejoides* Coss.

### 1.1. Environmental and Human Factors Shaping Phytochemical Variation in MAP

Phenotypic variation in the yield and composition of secondary metabolites (phytochemicals) within a species in different locations can be driven by genetic differences in plant populations in different sites or be influenced by differences in environmental factors. A number of studies have investigated the drivers of phytochemical variation, including in *Thymus* spp. [12,13], *Origanum syriacum* L. [14], *Camellia sinensis* (L.) Kuntze [5], *Monarda fistulosa* L. [15], *Mentha suaveolens* Ehrh. [12], and *Tanacetum vulgare* L. [16]. El-Alam et al. [14] showed a unique oil “chemotype” for *Origanum syriacum* L. in different locations, and they suggest chemical component biosynthesis pathways varied depending on the edaphic conditions. Many studies have found that geographic variation is associated with genetic rather than environmental differences between the locations [17,18,19]. Wolf et al. and Butcher et al. [16,17] suggest that the relative importance of genetic vs. environmental factors likely depends on the plant species and compounds under investigation. In a study of root extracts of *Echinacea angustifolia* grown from seed and collected from different wild sources in a controlled environment, Binns et al. [20] found a positive relationship between phytochemicals and latitude of wild plant populations from which the seed was obtained, but the directionality of the relationship varied from chemical to chemical. Additionally, human ecosystem alterations have been found to impact the chemical composition of plants [21].

### 1.2. Thyme and Thymus Saturejoides

Thyme species are key components of traditional herbal medicine in Morocco and are economically important, as essential oils are a key export. Thyme ranks second among Moroccan exported medicinal plants [22]. Rahmani [22] estimated that a mean of 1967 tons of dry thyme (all species) were exported from Morocco between 2002 and 2014. In 2021, national annual exports were estimated at 2.94M tons of thyme, with an export value for Morocco of USD 11.81M [23]. With the increased realization that some wild species of thyme are being overexploited, some are now experimenting with the farming of thyme species; however, the majority of thyme is still collected in the wild from 300,000 hectares of Moroccan forest [24]. Agadir, Marrakech, Essaouira, Beni Mellal, and Taroudant are the centers of national and international commerce in thyme. The sale prices of various types of thyme are different, depending on the provenance and rarity of the species in the market and in the wild. Associated with the growing importance of *Thymus* spp. in Morocco, there has been a lot of research on the chemical composition of different thyme species, but evidence on the environmental drivers of chemical composition remains limited.

There are 22 species and subspecies of thyme in Morocco [25], nine of which are endemic. Six thyme species among the twenty-two that exist in Morocco are traded and used as remedies for an extensive array of ailments, including antibacterial, antifungal, antioxidant, expectorant, mucolytic, and insecticidal properties, among others [26,27,28,29,30]. One Moroccan thyme species, *T. saturejoides* Coss., is listed as a vulnerable species in the IUCN Red List [31].

Each thyme species and subspecies has a different chemical composition; however, composition also varies greatly across populations and ecosystems within a given species. Certain species and plants from certain regions are preferred in the market over others. The thyme species that is most economically important and most appreciated in the herbal markets is the *T. saturejoides*, an endemic and vulnerable species with a range located in the Atlas Saharan, Anti Atlas, High Atlas, and Maamourra. *T. saturejoides* is typically found between 500 and 1500 m and 1500 and 2500 m. Two subspecies have been distinguished by Fennane [32], *Thymus saturejoides* subsp. *commutatus* Batt. and *T. saturejoides* subsp. *saturejoides*; however, according to the WFO Plant List [33], three subspecies have been recognized, including subsp. *commutatus* Batt., subsp. *saturejoides*, and subsp. *pseudomastichina* (Ball) Dobignard. *T. saturejoides* has been the subject of several studies examining essential oil yields and chemical diversity, but no studies to our knowledge have explored the drivers of chemical composition and diversity within the species. Thompson and Gilbert and Hudaib et al. [34,35,36] have worked extensively on factors that shape the chemical composition of *T. vulgaris* in southern France and in Sinai. These works have shown that climate and genetics, harvest period, vegetative cycles, soil, and exposure are all associated with the content of one or more chemical compounds.

Based on data extracted from the existing literature and studies, this paper examines the geographical distribution of the chemotype of *T. saturejoides* across Morocco. The mapping of *T. saturejoides*’s chemical composition allows us to examine different factors that shape chemical diversity and variation. Thyme, in general, and *T. saturejoides* as a case provide an interesting model to study how genetic and environmental variables and climate change influence the variation and the maintenance of secondary metabolites.

## 2. Results

### 2.1. Thymus Saturejoides’s Chemical Diversity and Variation

**Figure 1:**
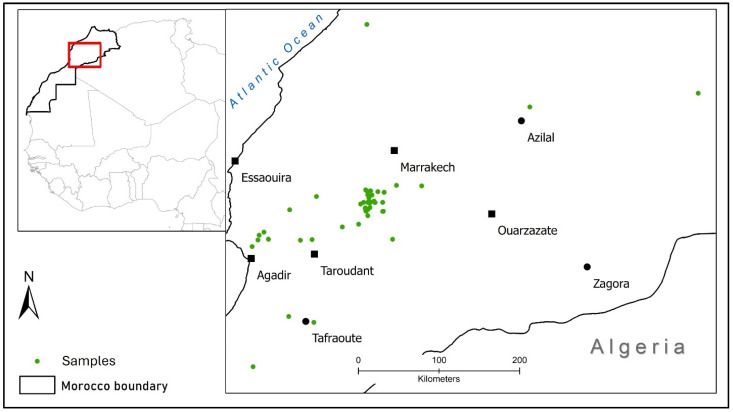
Map showing the distribution of *T. saturejoides* samples in Morocco from the papers reviewed.

The vast majority of the *T. saturejoides* samples in this study were distributed between the Marrakech and Taroudant regions (see Figure 1). The descriptive statistics of the dataset are reported in Table 1. Moroccan *T. saturejoides* has been characterized as having high concentrations of borneol, carvacrol, camphene, and thymol, as well as moderate levels of terpineol and low levels of other terpenes. The percentage of borneol concentration ranges from 7.5 to 59.37%, from 0 to 49.3% for carvacrol, from 0 to 27.4% for camphene, from 0 to 26.81% for thymol, and from 0 to 19.87% for terpineol. High carvacrol and thymol and lower borneol contents are associated with higher essential oil “quality” and value.

**Figure 2:**
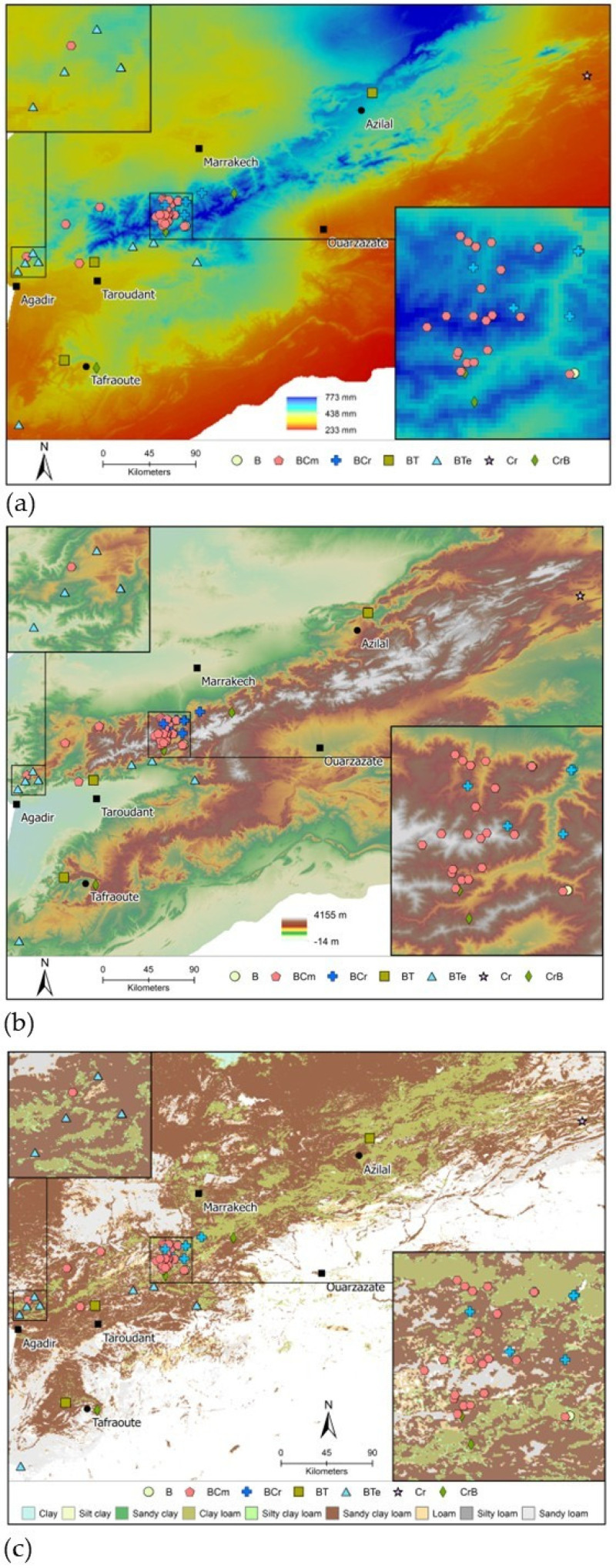
Overview of locations and chemotype (each chemotype is a unique color and shape). (**a**) Over a base map showing mean annual precipitation (mm) across 1993–2021 from the WorldClim; (**b**) soil type from Africa Soil Grids; and (**c**) elevation (m) from NASA’s Shuttle Radar Topography Mission (SRTM) from United States Geological Survey (USGS) Earth Explorer.

The majority of papers described chemical composition in terms of “Chemotype”, in addition to percent concentration of various chemicals. Chemotypes are determined based on which phytochemicals are present in the greatest amounts in a sample. Seven distinct chemotypes were reported for *T. saturejoides*, and these are reported in Table 2. The chemotype “BCm” (with high borneol and camphene) was the first most abundant chemotype and comprised approximately one-third of the samples. The geographic distribution of this chemotype is overlaid on maps of soil type, precipitation, and elevation, as shown in Figure 2. We did not detect significant relationships between environmental variables and chemotypes in our regression models (likely due to the very small N of all chemotypes other than “BCm”).

### 2.2. Environmental Factors and Individual Phytochemicals

**Figure 3:**
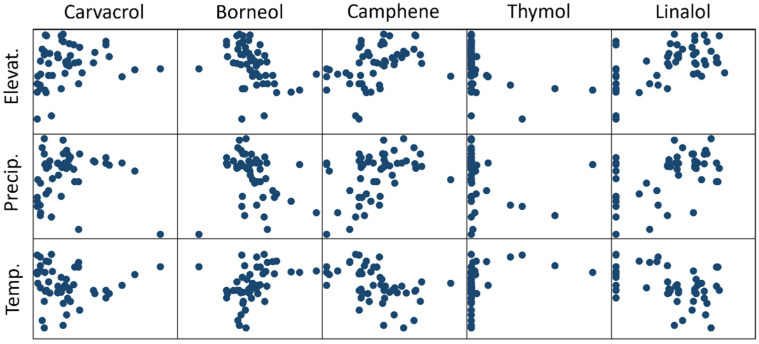
Scatter plot of environmental factors (elevation, precipitation, and temperature) versus individual chemicals (carvacrol, borneol, camphene, thymol, and linalool).

Our regression models examining the relationship between environmental variables and the percent concentration of key chemicals had a low predictive value (with the highest R-squared = 0.34 for linalool). Except for elevation, none of the other environmental variables were statistically significantly related to the percent concentration of any of the chemicals. Elevation was significant in the models for thymol (*p* = 0.030), linalool (*p* = 0.019), p-cymene (*p* = 0.001), carvacrol (*p* = 0.072), and borneol (*p* = 0.056). Scatter plots showing the relationships between these five key chemicals and environmental variables are shown in Figure 3. These scatter plots suggest that there is a relationship between precipitation and temperature and some of these chemicals, relationships that are not seen in regression models, likely because these other environmental variables covary with elevation. The scatter plots also suggest that some of the relationships between the environmental variables and the content of some of the chemicals might be nonlinear (we did not include nonlinear terms in the regression models because of concerns over sample size and power).

### 2.3. Principal Component Analysis (PCA) of Chemical Composition

To test if the chemotype identified in the literature adequately describes the variation between samples, we ran a principal component analysis (PCA) on the chemical composition of the samples included in the dataset (Table 3). The PCA identified components that were only partially related to the chemotype reported in the literature. The chemotypes in the literature focus on the most dominant phytochemicals and are, thus, largely driven by borneol. Those identified through PCA focus on phytochemicals, with the most (unique) variation with thymol, camphene, linalool, carvacrol, and borneol being the chemicals with the most variation. Coincidentally, these may in fact better represent essential oil quality, which is believed to be driven by carvacrol and thymol contents.

The variation in Component 1 depended on just a few samples that have high thymol and other chemicals (carvacrol methyl ether, tricyclene, β-myrcene, β-pinene, and α-thujene). Samples that load strongly onto Component 1 occurred at lower elevation and low precipitation and very high temperature. Component 2 was shaped by variation in camphene, linalool, α-pinene, and others ((E)-caryophyllene, α-pinene, and delta-3-carene). A lot of samples that loaded strongly onto Component 2 occurred at the lower temperatures. Component 3 was shaped by variation in carvacrol (low), borneol, and others (α-terpineol, linalool, γ-terpinene, and p-cymene).

**Figure 4:**
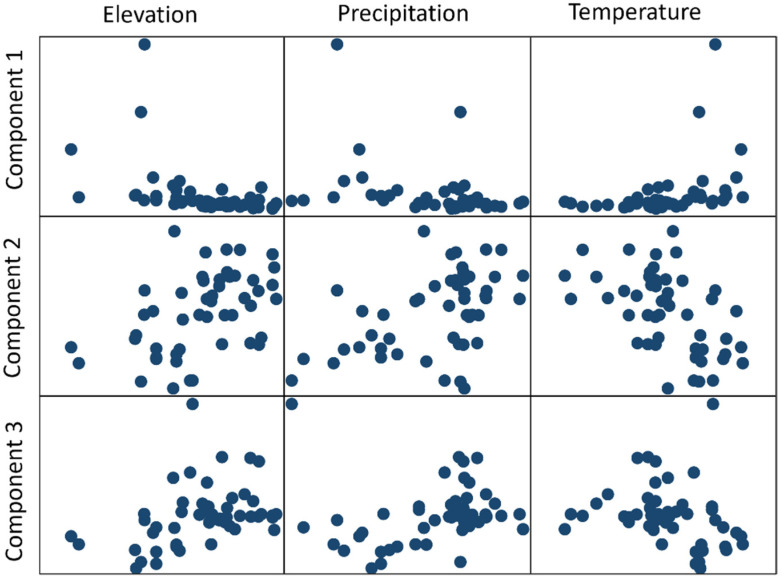
Principal component analysis (PCA) versus environmental factors (elevation, precipitation, and temperature).

We looked at the relationship between the components identified with the PCA and the environmental variables, and we found clear relationships (scatter plots are shown in Figure 4). Comp 1 (characterized by high thymol) occurred in low elevation (correlation r = −0.452), low precipitation (correlation r = −0.377), and high temperature settings (correlation r = 0.374). Comp 2 (characterized by high camphene and linalool) occurred at low temperatures (correlation r = 0.472), high precipitation (correlation r = 0.451), and high elevation (correlation r = −0.496). Comp 3 (characterized by high carvacrol and low borneol) occurred at moderate temperatures, moderate precipitation, and moderate-to-high elevation (around 1000 m) (correlation r = 0.388). We also ran linear regression models, and as with other regressions, only one factor remained significant (for Component 1 and Component 3, it was elevation, and for Component 2, it was temperature).

## 3. Discussion

Other research has shown that elevation plays an important role in the relative amount of flavonoids in other *Thymus* species [37]. *Thymus* plants growing at high altitude and under mesic conditions produce a greater relative amount of flavonoids in comparison to those grown at low altitude and in semi-arid conditions. The authors of this study explain these differences as physiological adaptations to protect the plant against extreme conditions [37]. Another study has shown a distinct chemical composition of *T. vulgaris* at different elevations in Catalonia. In this study, linalool yield increases with the elevation, whereas 1,8-cineole was highest at lower elevations [38]. Gherairia et al. [39] have shown that elevation has an important impact on the biosynthesis of terpenoids and that the anabolism of hydrocarbon monoterpenes is favored at high elevation, while that of oxygenated monoterpenes is favored at low elevation. El-Jalel et al. [40] showed that the variation in elevation shapes the heterogeneity in the composition of the *T. capitatus* in Libya.

Although we did not find an association between chemical composition and temperature and precipitation, this does not exclude the possibility that a relationship exists at the finer spatial or temporal scales. The broad time scale of the spatial datasets we used for temperature and precipitation (28-year mean) may have limited our ability to detect associations between these and chemical compositions. Year-, season-, and even day of harvest-specific precipitation and temperature variations could shape the phytochemical content.

Other research has shown that the different chemotypes of *T. vulgaris* have differential sensitivity to the cold, with the phenolic chemotype (thymol and carvacrol) being more sensitive to freezing (temperatures below zero degrees Celsius) and the non-phenolic chemotype (mostly geraniol and linalool) being more freezing tolerant [41]. Thompson et al. [41] and Franks et al. [42] indicate that warming temperatures have led to shifts in the geographical distribution of freezing-sensitive chemotypes in *Thymus vulgaris*, which suggests that these traits are largely driven by plant genetics. Similarly, Amiot et al. [43] found that non-phenolic chemotypes had better survival and regrowth after early winter frost (−100 in early December) compared to phenolic chemotypes. Thompson et al. [41] showed that the lack of severe winter frost was associated with an increase in the frequency of the phenolic chemotype, highlighting the rapid phenotypic response of some species to climate change. Thompson et al. note that the increasing “occurrence and frequency of extreme climate events may have a profound influence on the spatial distribution of thyme chemotype” [44]. Other factors that may affect the spatial distribution of the species’ chemotypes and compositions may include sex ratio [45] and human activity [46,47]. For example, Eriksson et al. [46] showed that the distribution and dispersal of *T. serpyllum* is dependent on human activities, especially animal grazing.

Aside from the few above examples, most studies of the potential impacts of climate change on thyme and other MAP in Morocco have focused on the effect of soil salinization and water stress on the physiology of different species [48,49,50] or range shifts expected under future climate change scenarios, e.g., in [38,51,52,53]. The IUCN has noted that most wild-collected MAPs, including thyme species, are fairly widespread and located at lower altitudes, making them less vulnerable to climate change compared to other plants with narrower ecological requirements [54]. Yet, climate change is already driving changes in physiological traits for a wide variety of medicinal plant taxa [55]. Given our findings on environmental factors, especially elevation, shape, and chemical composition of *T. saturejoides*, it is likely that climate change will impact the chemical composition of this species and/or change the distribution of chemotypes. Impacts on the chemical properties of medicinal plants are an important pathway through which climate change will impact human health—changing the medical properties and economic value of key plant resources.

## 4. Materials and Methods

### 4.1. Systematic Literature Review and Data Extraction

A literature review was carried out to create a dataset of chemical composition in *T. saturejoides* collected at different sites across Morocco. We searched on Web of Science and Google Scholar to identify any papers that included data on the chemical composition of *T. saturejoides*. The key words used included the following: “Morocco”, “chemotype”, “ecotypes”, “*Thymus satureioides*”, “*Thymus saturejoides*”, “polymorphism”, “oil”, “ethnopharmaology”, “thymol”, “carvacrol”, “borneol”, “terpineol”, “camphene”, “environment”, and “climate”. We used both specific names, *saturejoides* Coss. and Balansa and *satureioides* Coss. and Balansa, to track papers that were published before the specific name was changed to *saturejoides*. Additionally, we reviewed the reference lists of included papers to identify additional papers.

Peer-reviewed studies as well as the grey literature were included. We included papers in French and English, with no restriction on the date of publication. We selected only papers that included primary data on chemotype or plant chemistry. Studies with mixed objectives that include chemical analysis and microbiology, pharmacology, or other aspects were included in the review. Experimental medical research papers that focus on the use of extracts or essential oils but not include chemical composition information were excluded. We also only included papers that reported data specific to *T. saturejoides* and with clear information on where the plants came from.

Our search in Google Scholar and the Web of Science generated 406 sources. The majority of these were then excluded from the study. The most common reasons for exclusion were as follows: papers that listed plant species and their uses in treating different illnesses; papers that focused on multiple species of thyme without data specific to *T. saturejoides*; and papers that lacked location information. Many papers did not even provide clear information on whether the reported chemical composition data were from primary or secondary data. After screening, a total of 15 sources and 51 samples met the inclusion criteria.

From each of the included papers, we extracted key information, including the chemical composition, the location of harvest, the elevation, the date of the study, etc. We extracted data on the content of carvacrol, borneol, camphene, thymol, α-terpineol, linalool, γ-terpinene, p-cymene, (E)-caryophyllene, α-pinene, carvacrol methyl ether, tricyclene, β-myrcene, β-pinene, α-thujene, (e)-β-caryophyllene, α-terpinyl acetate, delta-3-carene, and β-caryophyllene as a percentage concentration of the essential oils obtained, assessed using a gas chromatography (GC/MS) analysis in the majority of the studies.

### 4.2. Environmental Variables

Using the geographical location of plant harvests listed in each paper (Figure 1), we determined an x-y GPS location. We used the x-y point to determine the elevation (if not listed in the paper), the mean annual temperature across 1993–2021, the mean annual rainfall/precipitation across 1993–2021, the soil type, and the soil carbon from publicly available spatial datasets (Table A1). The soil data were accessed and downloaded from Africa Soil Grids, and the soil texture was extracted from the International Soil Reference and Information Centre (ISRIC) dataset (textural class (USDA) of the soil fine earth fraction, aggregated over a rootable depth and the top 30 cm, mapped at 1 km resolution, available at [56]). The elevation is extracted from NASA’s Shuttle Radar Topography Mission (SRTM) elevation (downloaded from the United States Geological Survey (USGS) Earth Explorer website in [57] at a resolution of 1 arc-second). The annual mean temperature and precipitation means were between 1970 and 2000 at a resolution of 30 s (~1 km^2^) from the WorldClim database [58], released in 2020 and accessed in September 2022 [59]. All of the data were processed in ArcMap 10.8.2.

### 4.3. Statistical Analysis

We calculated the descriptive statistics for the variables in the new dataset. We then ran linear regression models to determine if any of these environmental variables were associated with the percent concentration for each of the following chemicals: carvacrol, borneol, camphene, thymol, α-terpineol, linalool, p-cymene, and α-pinene. We also ran logit regression models to examine the association between the environmental variables and the different “chemotypes”, as identified in each paper. Finally, we used a principal component analysis (PCA) of the chemical compositions to identify data-driven “components”, and we examined the relationship between these and environmental variables with regression models. Given evolving approaches to *p*-values and the small sample size of this study, we elected to use *p* < 0.1 as our benchmark for statistical significance.

## 5. Conclusions

There is quite a bit of literature on *T. saturejoides* (including the ecology, physiology, conservation, and marketization), but noticeably less on the population composition in terms of phenotype and genetics. The current state of knowledge offers very little insight into the co-evolution of the social and ecological systems of *T. saturejoides* habitats. This paper contributes to filling this gap by improving our knowledge of the ways environmental factors shape *T. saturejoides* phenotypes in terms of chemical composition. This basic understanding is an important first step in designing future research that will examine the ways humans influence this economically important plant species through both human-induced climate change and more local human modification of ecosystems. Future research on how plant collectors and adjacent communities are interacting with and shaping *T. saturejoides* populations and genetics is another crucial gap highlighted by this work.

## Figures and Tables

**Table 1:** Descriptive statistics for data extracted from 15 papers and 51 samples.

	Min	Max	Mean	SD
Latitude	29.21425	32.26666	30.93690	0.468
Longitude	−9.57455	−4.59999	−8.38886	0.787
Elevation (meters)	247	2181	1483.5	451
Mean Precipitation (mm/yr)	148	617	440	110
Mean Temp (°C)	6.7	18.24	13.43	2.725
Carvacrol (%)	0	45.3	10.33	9.903
Borneol (%)	7.5	59.37	31.18	8.409
Camphene (%)	0	27.4	10.83	5.829
Thymol (%)	0	26.81	1.5	4.892
α-Terpineol (%)	0	19.87	6.85	5.392

**Table 2:** Chemotypes reported for *T. saturejoides*.

Chemotype	Chemical Traits	Sample Size (N)	Notes/Where It Is Commonly Found
B	High borneol	1	Commonly found in the Tiznit region
BCm	High in borneol and moderately in camphene	26	Mostly found in Ijoukak, Agoundis, and Imintanoute
BCr	High in borneol and carvacrol	7	Located in Asni, Ouirgane, and Agoundis
BT	High in borneol and thymol	3	Found in the Azilal, Bin Ouidane, and Taroudant regions
BTe	High in borneol and α-terpineol	9	Predominantly found in Imouzzar Ida Outanante
Cr	High in carvacrol	1	Found in Tafraout and Midelt
CrB	High in carvacrol and low level of borneol	4	Found Idni, Setti Fatma, Tafraout and Midelt

**Table 3:** The three primary components (Comp) identified by PCA and the weighting of each chemical onto them (weighting greater than ±0.3 highlighted in gray).

Variable	Comp1	Comp2	Comp3
carvacrol	−0.0579	−0.1702	0.4853
borneol	0.0162	−0.1256	−0.4179
camphene	−0.0860	0.4097	−0.0911
thymol	0.3172	−0.1027	−0.1249
α-terpineol	−0.1122	−0941	−0.3382
linalool	−0.0657	0.3964	0.0311
γ-terpinene	0.0009	−0.2163	0.4367
p-cymene	−0.0328	0.2896	0.3875
(E)-caryophyllene	−0.0046	−0.3579	0.1821
α-pinene	−0.0352	0.4313	0.0431
carvacrol methyl ether	0.3635	−0.0303	0.0503
tricyclene	0.4153	0.1024	0.0329
β-myrcene	0.4153	0.1024	0.0329
β-pinene	0.4153	0.1024	0.0329
α-thujene	0.4153	0.1024	0.0329
(E)-β-caryophyllene	0.0106	−0.0781	0.1890
α-terpinyl acetate	0.0569	−0.1067	−0.1358
delta-3-carene	−0.1111	0.3145	0.0629
β-caryophyllene	0.1722	−0.0834	−0.0908

## Data Availability

The data that support the findings of this study are available from the corresponding author upon reasonable request.

## Appendix A

**Table A1:** Data for each sample used in the analysis and the original source of the data.

Latitude	Longitude	Chemotype	Yield (%)	Altitude (m)	Soil Carbon (%)	Soil Texture *	Rainfall (mm)	Temperature (°C)	References
30.90000	−8.28333	CrB	2.58 ± 0.03	1670	23	6	487	12.1	[60]
31.23333	−7.68333	CrB	2.40 ± 0.03	2018	30	6	496	12.6	[61]
29.77541	−9.16638	BT	2.66 (Av)	940	14	6	240	16.5	[28]
30.68202	−9.49552	BCm	1.7 ± 0.4	936	17	6	334	14.6	[62]
30.95000	−8.30000	CrB	2.40 ± 0.03	1937	18	6	524	11.4	[29]
32.26666	−4.59999	Cr	2.07 ± 0.05	1392	5	6	148	16.3	[29]
30.80588	−8.38700	BTe	2.7	1093	12	6	421	15.6	[63]
30.55588	−9.57455	BTe	1.85	210	10	6	233	18.2	[63]
30.62897	−9.50933	BTe	2.33	927	13	6	310	15.5	[63]
30.63716	−9.39225	BTe	2.28	1178	15	6	329	15.2	[63]
30.63758	−9.39297	BTe	1.94	1201	15	6	329	15.2	[63]
31.16777	−8.17250	BCr	--	1210	31	4	498	13.4	[64]
30.95000	−8.11000	B	1.65	2040	32	4	476	12.8	[65]
31.11481	−8.85653	BCm	--	1221	18	4	416	13.7	[66]
30.63400	−8.90542	BT	--	247	14	6	285	18.1	[67]
31.16777	−8.17250	BCm	1.05	1289	31	4	498	13.4	[68]
31.16277	−8.10083	BCr	1.02	1542	12	6	406	16.2	[68]
31.16138	−8.10361	BCr	1.04	1526	13	6	406	16.2	[68]
31.12944	−8.23722	BCm	1.59	1577	21	6	541	11.4	[68]
31.04861	−8.11777	BCr	0.65	1766	20	6	503	12.1	[68]
31.02999	−8.36527	BCm	--	1839	26	9	573	7.9	[68]
31.18833	−8.30777	BCm	0.48	1457	21	6	514	12.8	[68]
31.17777	−8.29416	BCm	0.98	1515	15	4	481	13.9	[68]
31.16944	−8.28055	BCm	0.67	1549	28	4	498	13.3	[68]
31.16944	−8.28055	BCm	0.44	1648	28	4	498	13.3	[68]
31.06305	−8.21527	BCr	0.78	1695	40	6	527	10.9	[68]
31.04777	−8.20333	BCm	0.2	1713	27	5	542	10.9	[68]
31.05055	−8.25222	BCm	0.37	1791	34	6	617	6.7	[68]
31.04138	−8.26277	BCm	1.47	2181	39	6	610	7.1	[68]
31.09694	−8.27138	BCm	0.66	1742	26	6	508	12.2	[68]
30.99000	−8.26027	BCm	0.7	2162	15	6	494	12.5	[68]
30.98055	−8.31361	BCm	0.86	2146	19	6	472	12.6	[68]
30.96777	−8.29638	BCm	0.97	1718	30	6	496	12.2	[68]
31.04888	−8.28500	BCm	1.67	1882	21	7	542	9.5	[68]
30.96583	−9.15555	BCm	0.38	1643	19	4	438	12.6	[68]
30.95333	−8.30666	BCm	1.47	2013	29	6	543	10.6	[68]
31.17694	−8.25388	BCm	0.85	1496	15	6	471	14.3	[68]
30.98694	−8.31138	BCm	1.4	2146	20	6	484	12.3	[68]
30.94833	−8.11805	BCm	2.29	1964	31	6	480	12.6	[68]
30.96916	−8.28388	BCm	0.9	1942	23	4	467	13.5	[68]
31.13305	−8.28527	BCr	0.81	1528	19	6	508	12.6	[68]
31.04916	−8.33194	BCm	--	1485	27	6	560	8.8	[68]
29.71014	−8.88659	CrB	1.78	1270	12	6	254	15.6	[69]
31.23955	−7.96436	BCr	--	1369	18	6	458	15.1	[70]
32.11373	−6.47671	BT	--	906	20	5	490	15.4	[70]
30.62583	−9.03619	BCm	2.02	1020	13	6	291	17.8	[71]
30.77411	−8.56908	BTe	1.35	850	13	6	346	17.2	[71]
29.21425	−9.56369	BTe	2.16	1050	--	--	172	16.7	[71]
30.63708	−8.00808	BTe	2.32	1240	19	6	362	17.1	[71]
30.71628	−9.44237	BTe	3.3 ± 0.02	1568	25	6	399	13	[72]

*: 1 = clay, 2 = silt clay, 3 = sandy clay, 4 = clay loam, 5 = silty clay loam, 6 = sandy clay loam, 7 = loam, 8 = silty loam, 9 = sandy loam.
